# Gut microbiome regulates inflammation and insulin resistance: a novel therapeutic target to improve insulin sensitivity

**DOI:** 10.1038/s41392-024-01746-y

**Published:** 2024-02-21

**Authors:** Dilvin Semo, Holger Reinecke, Rinesh Godfrey

**Affiliations:** Vascular Signalling, Molecular Cardiology, Department of Cardiology I-Coronary and Peripheral Vascular Disease, Heart Failure, University Hospital Münster, 48149 Münster, Germany

**Keywords:** Metabolic disorders, Inflammation

In a recent study published in *Nature*, Takeuchi et al. used a multi-omics strategy in humans to provide compelling evidence that increased carbohydrate metabolism by the gut microbiota contributes to insulin resistance (IR). The authors uncovered a previously unknown link between elevated gut monosaccharides and host inflammatory cytokines and suggest potential intervention opportunities to combat IR by targeting specific gut microbial species.^1^

Type 2 diabetes mellitus (T2DM) affects approximately 540 million people worldwide. Insulin-resistant cells cannot respond to insulin and use glucose from the blood, a critical aspect responsible for developing T2DM and metabolic syndrome (MetS). Combating IR reduces T2DM and T2DM-driven cardiovascular diseases (CVD). Several previous studies have shown an association between gut microbial carbohydrate metabolism and the development of obesity^2^ and pre-diabetes.^3^ However, these studies have not been able to provide a detailed mechanistic link mediating this association, particularly in humans. Using unbiased faecal metabolomics with metagenomics, host metabolomics and transcriptomics data from 306 individuals without T2DM, the authors profiled the involvement of the gut microbiome in IR or insulin sensitivity (IS). They also looked for associations between faecal metabolites and MetS.

Takeuchi and colleagues found an association between IR or MetS and increased levels of faecal monosaccharides. Host-accessible monosaccharides such as fructose, galactose, mannose and xylose were increased in people with IR and MetS. The excess monosaccharides could promote lipid accumulation and activate immune cells, leading to an increased pro-inflammatory cytokine response in the host. Remarkably, the authors were able to assign a specific inflammatory cytokine, metabolic and transcriptome signature to individuals with IR.

**Fig. 1:**
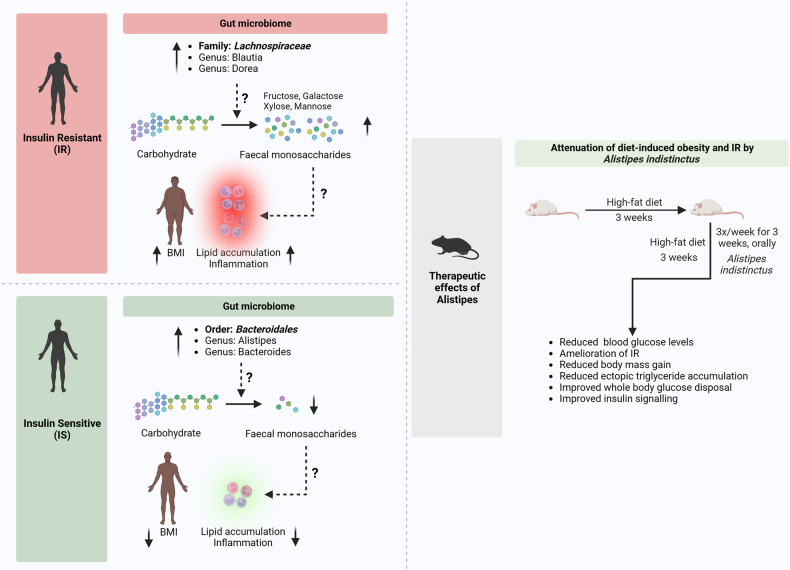
The importance of gut microbial metabolism in the regulation of insulin sensitivity in humans and mice. In insulin-resistant individuals, due to the enrichment of the bacteria belonging to the *Blautia* and *Dorea* genus of the *Lachnospiraceae* family in the gut, the carbohydrate metabolism gets altered, resulting in the accumulation of host-accessible faecal monosaccharides. The accumulated monosaccharides contribute to higher lipid accumulation, inflammation induction, and obesity promotion. These alterations collectively promote insulin resistance. In contrast, the Bacteroidales-type bacteria of the genus *Alistipes* and *Bacteroides* are enriched in the gut of insulin-sensitive individuals. Due to the difference in the metabolism of *Alistipes* and *Bacteroides* compared to *Blautia* and *Dorea*, the faecal monosaccharide accumulation is much reduced. This potentially contributes to lower lipid accumulation, inflammation and protection from obesity. Oral administration of *Alistipes indistinctus* in mice fed with a high-fat diet resulted in complete protection against insulin resistance and obesity. The figure has been created with BioRender.com

When the gut microbiota was characterised in detail, the *Lachnospiraceae* microbiome was associated with an excess of faecal monosaccharides. In addition, the increased presence of *Lachnospiraceae* (*Dorea*, *Blautia*) in the gut microbiota was positively correlated with IR. On the other hand, IR and faecal monosaccharides were lower in individuals who harboured more Bacteroidales-type bacteria in their gut, potentially highlighting the role of Bacteroidales-type bacteria (*Bacteroides, Alistipes*) in mediating IS. Further in vitro studies showed that Bacteroidales — particularly *Alistipes indistinctus* — could metabolise the monosaccharides accumulated in the faeces of people with IR. The application of these exciting findings was tested in mice. The mice were fed a high-fat diet and orally given the bacteria identified in the multi-omics analysis. Transfer of IS-associated bacteria (*Alistipes indistinctus*) reduced blood glucose and faecal monosaccharide levels, improved lipid accumulation and ameliorated IR. These data suggest a role for IS-associated microbiota in the treatment of IR (Fig. 1).

This study has direct implications for how insulin resistance is diagnosed and treated. Because of its association with IR, the presence of *Lachnospiraceae* bacteria in the gut could be a novel biomarker for pre-diabetes and IR. Similarly, treatment with probiotics containing *Alistipes indistinctus* could improve insulin sensitivity in pre-diabetic individuals.

Conceptualised as a single-centre trial at the University of Tokyo Hospital in Japan, human data from different ethnic backgrounds are lacking. The observed effects should be validated in further studies in different human cohorts worldwide to rule out the role of genetics and environment, such as the dietary habits of the study group. Former studies revealed drastic differences in the gut metagenome between European and Chinese cohorts,^4^ and it is essential to consider this aspect before making broader conclusions from this study.

By bringing new data to the metabolomics and IR field, the study by Takeuchi and colleagues raises many questions and allows us to outline three crucial aspects for further investigation.

Firstly, although *Alistipes indistinctus* helped alleviate IR, further experiments are needed to understand the specific pathways by which it affects host metabolism and regulates carbohydrate degradation. Therefore, further studies investigating potentially involved pathways or in vitro studies on the link between bacterial and host glucose metabolism are needed. While it is known that chronic inflammation causes various signalling abnormalities that lead to insulin resistance,^5^ the direct link and the mechanisms that mediate this effect remain to be elucidated. It also remains unclear whether there is a direct link between specific gut microbiota and IS. Lower levels of gut monosaccharides may reduce chronic inflammation in the host through unknown pathways. However, reduced intestinal monosaccharide levels may only be a novel biomarker for monitoring the IS phenotype.

Another important aspect is the need for a global analysis of the changes in insulin signalling in the major sites of glucose consumption, such as skeletal and adipose tissues other than the liver, which the authors focused on in this study. Further studies on metabolic and growth factor pathways involved in insulin signalling, e.g. ERK/MAPK and IRS/PI3K/AKT pathways, will allow a better understanding of the effects of *Alistipes indistinctus* on insulin signalling at a whole-body level.

Finally, this study allows us to discuss the impact of our diet and lifestyle on our cardiovascular risk profile and the known classic effects of diet, such as weight gain. If higher monosaccharide levels are associated with IR and inflammatory induction, does changing our dietary intake reduce this effect, regardless of the type of bacteria in our gut? This question could be answered in studies investigating the role of different gut microbiomes in animals on a strict diet; otherwise, this remaining question could also be addressed in a randomised, double-blind study with IR patients on a controlled low-glucose diet or a controlled high monosaccharide diet. Such studies could provide new insights into the effect of dietary intake on monosaccharide levels and inflammation in the host.

In conclusion, the study by Takeuchi and colleagues draws attention to the complex relationship between humans and their gut microbiomes and the role of microbial metabolism in the development and cause of IR. Although the study has limitations in elucidating the exact mechanisms by which IS-associated bacteria could reduce inflammation and alleviate IR, it provides relevant insights into the symbiosis between the host and gut microbiome in the context of IR pathogenesis. This study revealed better ways detect IR and demonstrated the potential impact of IS-associated probiotics in mitigating IR. Further investigation, including human clinical trials, is needed to determine whether modifying the gut microbiome could be a viable therapeutic strategy in primary IR prophylaxis or alleviating IR and IR-associated diseases.
